# Small Mouse Islets Are Deficient in Glucagon-Producing Alpha Cells but Rich in Somatostatin-Secreting Delta Cells

**DOI:** 10.1155/2016/4930741

**Published:** 2016-07-18

**Authors:** Joey Lau, Eva Grapengiesser, Bo Hellman

**Affiliations:** Department of Medical Cell Biology, Uppsala University, Box 571, 751 23 Uppsala, Sweden

## Abstract

Small and big mouse islets were compared with special reference to their content of glucagon-producing *α*-cells and somatostatin-producing *δ*-cells. Areas stained for glucagon and somatostatin were measured in the largest cross section of small (diameter < 60 *μ*m) and big (diameter > 100 *μ*m) islets. Comparison of the areas indicated proportionally more *δ*- than *α*-cells in the small islets. After isolation with collagenase these islets were practically devoid of *α*-cells. We evaluated the functional importance of the islet size by measuring the Ca^2+^ signal for insulin release. A majority of the small islets responded to the hyperpolarization action of somatostatin with periodic decrease of cytoplasmic Ca^2+^ when glucose was elevated after tolbutamide blockade of the K_ATP_ channels.

## 1. Introduction

Studies of the endocrine cells in pancreas face special problems dependent on their distribution into islets containing several types of hormone-producing cells. Postnatal growth of the endocrine pancreas is strictly regulated [1, 2]. Independent of age, the total volume of the endocrine pancreas is symmetrically related to the diameter of the islets in various species including mouse [3] and man [4, 5]. Accordingly, a majority of the endocrine cells are situated in the medium-sized and not in the numerous small islets [6, 7]. 

A key question is how the size of the islets is related to their content of different types of hormone-producing cells. Increase of glucose is a more effective stimulator of insulin release from small rather than from big islets [8]. This observation can be attributed to several factors, including a higher proportion of *β*-cells. Originally the glucagon-producing *α*-cells were considered to be located essentially in small islets [9]. Today we know that a majority of the *α*-cells in most species appear in the periphery of big and medium-sized islets [8, 10, 11]. Current knowledge of the cytoarchitecture of small islets is based on studies of islets with a diameter less than 100–150 *μ*m [12, 13]. This size limit is less appropriate by also including medium-sized islets that account for most of the volume of the endocrine pancreas in rats [14], mice [3], and man [4, 5].

The aim of the present study was to compare the cytoarchitecture of small and big mouse islets in both sections of pancreas and after isolation with collagenase. The results indicate differences between small islets and big islets with regard to both proportions of *α*- and *δ*-cells and the Ca^2+^ oscillations induced by glucose in the absence of functional K_ATP_ channels.

## 2. Material and Methods

The experimental procedure was approved by the Animal Ethics committee of Uppsala University. Pancreas was removed from 3-4-month-old C57BL/6 mice allowed free access to food and water. The islets were studied either when situated in pancreas or after collagenase isolation followed by 1-2 days' culture. The procedure of isolation was similar to that of Lacy and Kostianovsky [15]. Pancreases were fixed in 10% (vol/vol) formalin and embedded in paraffin. Isolated islets were fixed in 4% (vol/vol) paraformaldehyde followed by embedding in agarose and finally paraffin [16].

### 2.1. Immunohistochemistry

The paraffin-embedded tissue was consecutively sectioned (5 *μ*m) and mounted on glass slides. Pancreas sections were double stained for glucagon (mouse monoclonal; Abcam, ab10988) and insulin (guinea pig polyclonal; Fitzgerald, Concord, MA, USA) followed by detection with MACH 3 polymer system (Biocare Medical, Concord, CA, USA) and developed with Vulcan Fast Red and 3,3′-diaminobenzidine (DAB), respectively [17]. Highly purified secondary antibodies (donkey anti-rabbit alkaline phosphatase (AP) and donkey anti-guinea pig horseradish peroxidase (HRP); Jackson ImmunoResearch Laboratories, West Grove, PA, USA) were used to prevent cross-reactivity during double staining of pancreas with somatostatin and insulin, and development with Vulcan Fast Red and DAB, respectively.

Sections of isolated islets were either immunostained for glucagon or somatostatin followed by detection with MACH 3 polymer systems and developed with Vulcan Fast Red or DAB, respectively. The immunostained sections were counterstained with hematoxylin.

**Figure 1:**
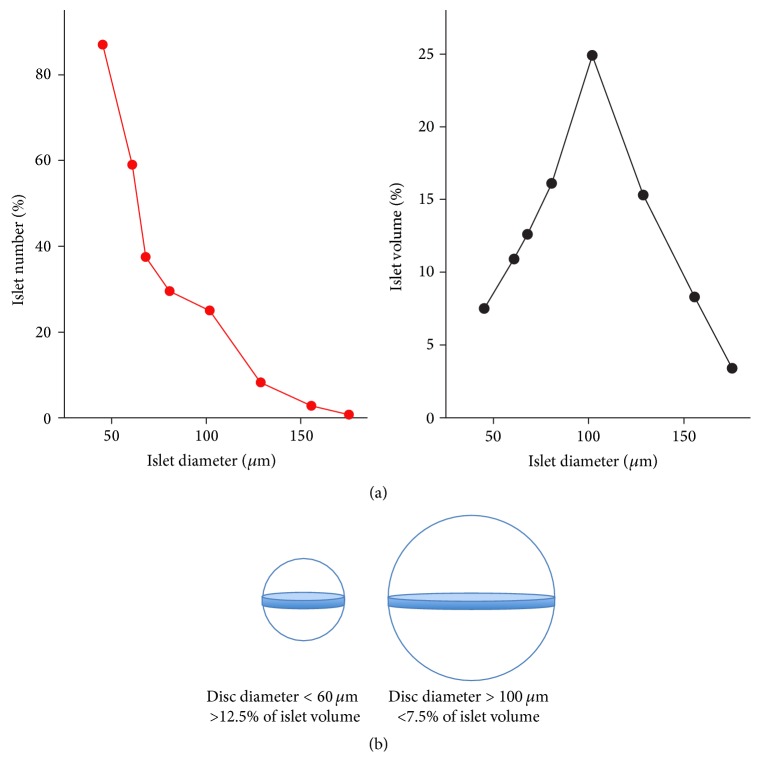
(a) Relations between islet diameter and the number (left panel) and total volume (right panel) of mouse islets. Redrawn from Hellman et al. [3]. (b) The largest cross sections were used for measuring the areas of small and big islets stained for glucagon and somatostatin. The contribution of a 5 *μ*m disc to the total volume of the islet is illustrated.

Previous observations on how the number and total volume of mouse islets are related to the islet diameter are shown in Figure 1(a). Referring to these data a comparison was made of the cellular composition in small (diameter < 60 *μ*m) and big (diameter > 100 *μ*m) islets. Areas of glucagon and somatostatin were measured in the largest cross section of the islets with a Leica LMD6000 microsystem (objective 40x). The experimental procedure made it possible to calculate the total areas of *α*- and *δ*-cells in islet sections but only to estimate their contributions to the islet volume. Observations in the largest cross section are more representative for small rather than big islets (Figure 1(b)). Even more important, peripherally located cells will contribute more to the islet volume in small rather than in big islets.

The functional importance of the islet size was evaluated by studying the glucose generation of cytoplasmic Ca^2+^ oscillations mediated by the K_ATP_ channel-independent pathway. The concentration of cytoplasmic Ca^2+^ was measured with ratiometric fura-2 technique as previously described [18].

### 2.2. Statistical Analysis

All values are given as means ± SEM. Probabilities of chance differences between the groups were calculated using Student's unpaired two-tailed* t*-test. For all comparisons, a *P* value < 0.05 was considered statistically significant.

## 3. Results

**Figure 2:**
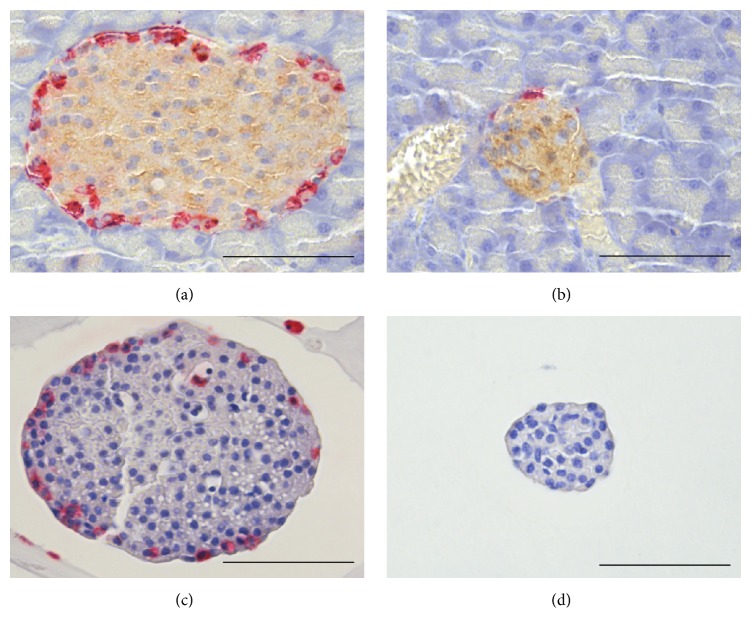
Double immunostaining of a big islet (a) and a small islet (b) in pancreas for glucagon (red) and insulin (brown). Immunostaining of an isolated big islet (c) and an isolated small islet (d) for glucagon (red). The isolated small islet (d) is devoid of glucagon-positive cells. Scale bars represent 50 *μ*m.

**Figure 3:**
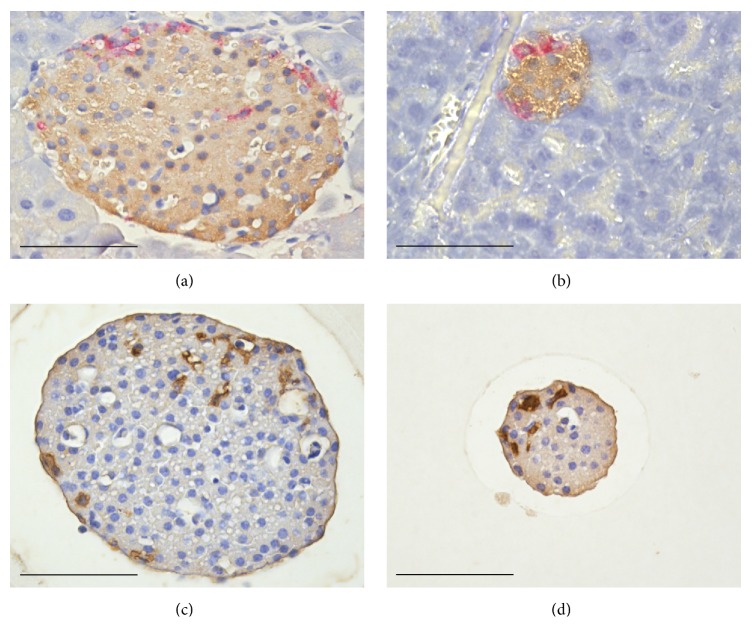
Double immunostaining of a big islet (a) and a small islet (b) in the pancreas for somatostatin (red) and insulin (brown). Immunostaining of an isolated big islet (c) and an isolated small islet (d) for somatostatin (brown). The somatostatin-producing *δ*-cells are located in both the core and periphery of the islets. Scale bars represent 50 *μ*m.

The topographic distribution of *α*- and *δ*-cells in small and big islets is illustrated in Figures 2 and 3. Both types of cells occurred in the islet periphery, often forming a mantle around a core with *β*-cells. In the big islets, *δ*-cells were located close to the capillaries also in the central core. The peripheral location was particularly pronounced for *α*-cells, whereas the *δ*-cells were intertwined between *β*- and *α*-cells. The difficulties to evaluate the proportions of *α*- and *δ*-cells in islets of different sizes are illustrated in Figure 1(b). The approach with measurements in the largest optical cross section will underestimate the number of *α*-cells in big islets due to their peripheral location. Nevertheless, the glucagon-positive area was twice as large (*P* < 0.001) in big compared with the small islets in pancreas sections (Table 1). The opposite was found for the area of somatostatin, which was larger in small rather than in big islets (*P* < 0.05).

Results from measurements in isolated islets are included in Table 1. In the big islets the percentage contribution of the glucagon area was 1.43 ± 0.28 and the small islets were practically devoid of *α*-cells. In the latter case, only 2 of 52 islets were positive for glucagon. A corresponding analysis revealed that the somatostatin area was larger in small rather than in big islets (*P* < 0.001).

**Figure 4:**
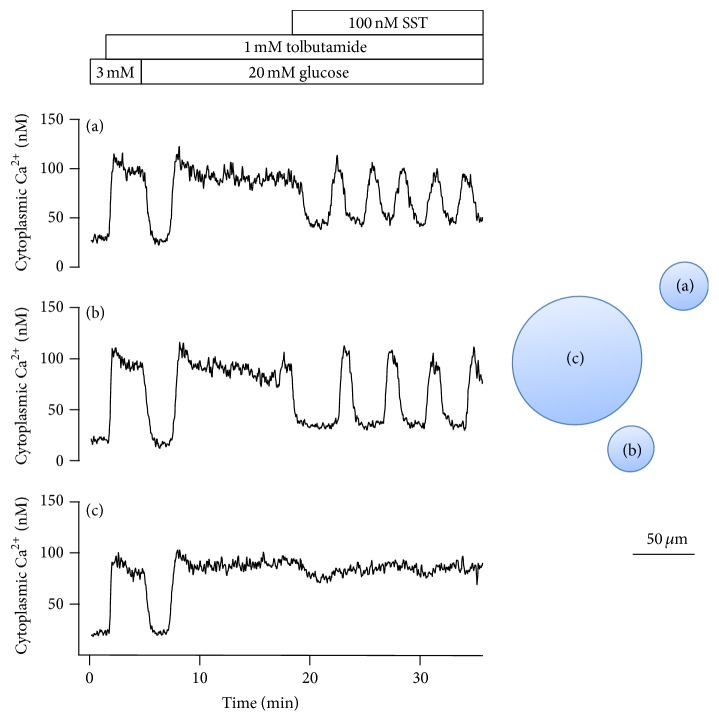
Effects of 100 nM somatostatin on the cytoplasmic Ca^2+^ concentration in one big and two small islets after elevation of glucose in the presence of 1 mM tolbutamide. Exposure to somatostatin resulted in glucose-dependent oscillations mediated by periodic interruption of elevated Ca^2+^ in 42 of 47 of the small islets and in 4 of 13 of the big islets.

Attempts were made to decide whether the oscillatory Ca^2+^ signal for glucose-induced insulin release was affected by the islet size. It was found that hyperpolarization with 100 nM somatostatin usually resulted in glucose-dependent Ca^2+^ oscillations in small islets after sulfonylurea blockade of the K_ATP_ channels (Figure 4). The oscillations appeared not only in the presence of 20 mM glucose but also at 8 or 5 mM. Taken together, the exposure to somatostatin resulted in glucose-dependent oscillations in 89% (42 of 47) of the small and 31% (4 of 13) of the big islets (*P* < 0.05).

## 4. Discussion

A reliable but tedious way to study the cellular composition of islets is to measure the area of different types of cells in consecutive thin sections. In the present study the areas stained for glucagon and somatostatin were determined in the largest cross section of the islets. This procedure makes it possible to get a sufficient number of data from small and big islets located in pancreas or isolated with collagenase. Including both the central core and the peripheral mantle of the islets, the approach provides an indirect estimate of the whole islet. It is important to note that the data presented underestimates the contribution of the peripherally located cells, essentially *α*-cells.

The size distribution of the islet volume is essentially symmetrical in birds [19], rodents [3, 4, 14], and man [4, 5]. A major part of the endocrine mouse pancreas is insulin-producing *β*-cells, the number of which usually increases when required for glucose homeostasis [20]. In the obese-hyperglycemic syndrome the increase results in 10 times more *β*-cells with maintenance of the symmetrical volume relation between the big and small islets [3]. In human diabetes the symmetry is maintained [21] despite a reduced islet mass [22]. The present study indicates that the glucagon-positive area is more than twice as large in big compared with small islets situated in the pancreas. Further evidence that big islets contain more *α*-cells was obtained from studies of islets isolated with collagenase and kept in culture for 1-2 days. In this case the small islets were practically devoid of *α*-cells but rich in *δ*-cells. The reason why isolation of islets results in a lower content of *α*-cells is open for discussion. It is possible that the peripheral location makes the *α*-cells particularly vulnerable to the isolation process [23].

Somatostatin-producing *δ*-cells are topographically related to insulin-producing *β*-cells in several species including rodents [19]. We now confirm the reports that *δ*-cells are located not only in the periphery but also close to the capillaries penetrating into the central part of the big islets [20, 24]. The area stained for somatostatin is proportionately larger in small rather than in big mouse islets. The difference was particularly pronounced after collagenase isolation.

The finding that small islets are rich in somatostatin-producing *δ*-cells but practically devoid of *α*-cells raises the question how this affects the oscillatory Ca^2+^ signal for insulin release. Periodic entry of Ca^2+^ can be generated not only by elevation of glucose followed by closure of K_ATP_ channels and depolarization. In the K_ATP_ channel-independent alternative, the high membrane resistance makes the *β*-cells sensitive to hyperpolarization resulting in oscillations due to periodic interruption of the entry of Ca^2+^. Somatostatin is one of several G-protein coupled agonists known to hyperpolarize *β*-cells [25]. We now report that exposure to somatostatin in the presence of sulfonylurea blockade of the K_ATP_ channels promotes glucose-dependent oscillations more often in small rather than in big islets. Similar effects were seen after hyperpolarization obtained by activation of the *α*_2_-adrenergic receptors (unpublished data).

Kilimnik et al. [22] have reported that the proportion of *α*-cells is higher in large rather than in small islets also in humans. Moreover, these authors observed preferential loss of large islets in patients with type-2 diabetes. Accordingly, the reduction of islet mass in diabetes results in a proportionally larger decrease of glucagon-producing *α*-cells than of *β*- and *δ*-cells, a situation supposed to counteract hyperglycemia [26]. Our observation that small islets are prone to oscillate in response to glucose provides additional support for the clinical importance of the islet size.

## 5. Conclusions

Big islets situated in the pancreas contain proportionally more *α*-cells than small islets. In contrast, small islets situated in the pancreas contain proportionally more *δ*-cells than big islets. This difference was even more pronounced in isolated islets. Moreover, isolated small islets are practically devoid of *α*-cells but rich in *δ*-cells. Our Ca^2+^ measurements revealed that exposure to somatostatin in the presence of sulfonylurea blockade of the K_ATP_ channels promoted glucose-dependent oscillations more often in small than in big isolated islets.

## Figures and Tables

**Table 1:** Proportions of *α*- and *δ*-cells in small and big islets. Areas stained for glucagon and somatostatin in the largest cross section of small (<60 *µ*m) and big (>100 *µ*m) islets. The data refer to observations made in paraffin sections of pancreas and isolated islets. All values are given as means ± SEM. ^*∗*^ *P* < 0.001 compared with small islets stained for glucagon in the pancreas. ^#^ *P* < 0.05 compared with small islets stained for somatostatin in the pancreas. ^†^ *P* < 0.05 compared with small isolated islets stained for glucagon. ^¤^ *P* < 0.001 compared with small isolated islets stained for somatostatin.

	Islets in pancreas		Isolated islets	
	Small islets	Big islets	Small islets	Big islets
Glucagon				
Number of animals	5	5	6	4
Number of islets	52	65	52	44
Area stained for glucagon (%)	3.22 ± 0.51	8.61 ± 0.50^*∗*^	0.1	1.43 ± 0.28^†^

Somatostatin				
Number of animals	5	5	4	5
Number of islets	68	51	35	50
Area stained for somatostatin (%)	4.60 ± 0.48	3.37 ± 0.22^#^	5.71 ± 0.58	3.54 ± 0.24^¤^

## Abbreviations

ATP: Adenosine triphosphate
DAB: 3,3′-Diaminobenzidine
AP: Alkaline phosphatase
HRP: Horseradish peroxidase.

## References

[1] Hellman B. (1959). The effect of ageing on the total volumes of the A and B cells in the islets of Langerhans of the rat. *Acta Endocrinologica*.

[2] Khadra A., Schnell S. (2015). Development, growth and maintenance of *β*-cell mass: models are also part of the story. *Molecular Aspects of Medicine*.

[3] Hellman B., Brolin S., Hellerstrom C., Hellman K. (1961). The distribution pattern of the pancreatic islet volume in normal and hyperglycaemic mice. *Acta Endocrinologica*.

[4] Hellman B. (1959). Actual distribution of the number and volume of the islets of langerhans in different size classes in non-diabetic humans of varying ages. *Nature*.

[5] Kaihoh T., Masuda T., Sasano N., Takahashi T. (1986). The size and number of Langerhans islets correlated with their endocrine function: a morphometry on immunostained serial sections of adult human pancreases. *Tohoku Journal of Experimental Medicine*.

[6] Jo J., Choi M. Y., Koh D. S. (2007). Size distribution of mouse Langerhans islets. *Biophysical Journal*.

[7] Jo J., Hara M., Ahlgren U., Sorenson R., Periwal V. (2012). Mathematical models of pancreatic islet size distributions. *Islets*.

[8] Farhat B., Almelkar A., Ramachandran K. (2013). Small human islets comprised of more *β*-cells with higher insulin content than large islets. *Islets*.

[9] Ferner H. (1952). *Das Inselsystem des Pankreas*.

[10] Alm G., Hellman B. (1964). Distribution of the two types of A-cells in the pancreatic islets of some mammalian species. *Acta Endocrinologica (Copenhagen)*.

[11] Dolenšek J., Rupnik M. S., Stožer A. (2015). Structural similarities and differences between the human and the mouse pancreas. *Islets*.

[12] MacGregor R. R., Williams S. J., Tong P. Y., Kover K., Moore W. V., Stehno-Bittel L. (2006). Small rat islets are superior to large islets in in vitro function and in transplantation outcomes. *American Journal of Physiology—Endocrinology and Metabolism*.

[13] Lehmann R., Zuellig R. A., Kugelmeier P. (2007). Superiority of small islets in human islet transplantation. *Diabetes*.

[14] Hellman B. (1959). The volumetric distribution of the pancreatic islet tissue in young and old rats. *Acta Endocrinologica*.

[15] Lacy P. E., Kostianovsky M. (1967). Method for the isolation of intact islets of Langerhans from the rat pancreas. *Diabetes*.

[16] Lau J., Mattsson G., Carlsson C. (2007). Implantation site-dependent dysfunction of transplanted pancreatic islets. *Diabetes*.

[17] Ullsten S., Lau J., Carlsson P.-O. (2015). Vascular heterogeneity between native rat pancreatic islets is responsible for differences in survival and revascularisation post transplantation. *Diabetologia*.

[18] Hellman B., Dansk H., Grapengiesser E. (2014). Activation of alpha adrenergic and muscarinic receptors modifies early glucose suppression of cytoplasmic Ca^2+^ in pancreatic *β*-cells. *Biochemical and Biophysical Research Communications*.

[19] Hellman B., Hellerström C. (1960). The islets of Langerhans in ducks and chickens with special reference to the argyrophil reaction. *Zeitschrift für Zellforschung und Mikroskopische Anatomie*.

[20] Kharouta M., Miller K., Kim A. (2009). No mantle formation in rodent islets-the prototype of islet revisited. *Diabetes Research and Clinical Practice*.

[21] Hellman B. (1961). The frequency distribution of the number and volume of the islets of Langerhans in man. 2. Studies in diabetes of adult onset. *Acta Pathologica et Microbiologica Scandinavica*.

[22] Kilimnik G., Zhao B., Jo J. (2011). Altered islet composition and disproportionate loss of large islets in patients with type 2 diabetes. *PLoS ONE*.

[23] Huang H., Novikova L., Williams S. J., Smirnova I. V., Stehno-Bittel L. (2011). Low insulin content of large islet population is present in situ and in isolated islets. *Islets*.

[24] Brereton M. F., Vergari E., Zhang Q., Clark A. (2015). Alpha-, delta- and PP-cells: are they the architectural cornerstones of islet structure and co-ordination?. *Journal of Histochemistry and Cytochemistry*.

[25] Braun M. (2014). The somatostatin receptor in human pancreatic *β*-cells. *Vitamins and Hormones*.

[26] Unger R. H., Cherrington A. D. (2012). Glucagonocentric restructuring of diabetes: a pathophysiologic and therapeutic makeover. *The Journal of Clinical Investigation*.

